# RETRACTED: Ghasemi Darestani et al. Association of Polyunsaturated Fatty Acid Intake on Inflammatory Gene Expression and Multiple Sclerosis: A Systematic Review and Meta-Analysis. *Nutrients* 2022, *14*, 4627

**DOI:** 10.3390/nu16193246

**Published:** 2024-09-26

**Authors:** Nutrients Editorial Office

**Affiliations:** MDPI, St. Alban-Anlage 66, 4052 Basel, Switzerland; nutrients@mdpi.com

The Journal retracts and amends the authorship of the article “Association of Polyunsaturated Fatty Acid Intake on Inflammatory Gene Expression and Multiple Sclerosis: A Systematic Review and Meta-Analysis” [1], cited above.

Following publication, concerns were brought to the attention of the publisher regarding the authenticity of the authorship of this article [1].

Adhering to our complaint procedure, an investigation was conducted by the Editorial Office and Editorial Board. Following communications with the authorship group and related institutions, the Editorial Office was unable to verify the identity, contribution, or affiliations of a number of the authors listed on this manuscript, nor could the origins of the study be confirmed. As a consequence, the Editorial Board has lost confidence in the integrity of the findings and has decided to retract this publication [1] as per MDPI’s retraction policy (https://www.mdpi.com/ethics#_bookmark30 (accessed on 29 August 2024)).

This retraction was approved by the Editor-in-Chief of the journal *Nutrients*.

The authors did not agree to this retraction.
